# Effects of Constant Flickering Light on Refractive Status, 5-HT and 5-HT2A Receptor in Guinea Pigs

**DOI:** 10.1371/journal.pone.0167902

**Published:** 2016-12-13

**Authors:** Bing Li, Xiumei Luo, Tao Li, Changyue Zheng, Shunmei Ji, Yuanyuan Ma, Shuangshuang Zhang, Xiaodong Zhou

**Affiliations:** 1 Central Laboratory, Jinshan Hospital affiliated to Fudan University, Shanghai, China; 2 Department of Ophthalmology, Jinshan Hospital affiliated to Fudan University, Shanghai, China; 3 The State Key Laboratory of Medical Neurobiology, the Institutes of Brain Science and the Collaborative Innovation Center for Brain Science, Shanghai Medical College, Fudan University, Shanghai, China; 4 Department of Dermatology, Jinshan Hospital affiliated to Fudan University, Shanghai, China; Bascom Palmer Eye Institute, UNITED STATES

## Abstract

**Purpose:**

To investigate the effects of constant flickering light on refractive development, the role of serotonin (i.e.5-hydroxytryptamine, 5-HT)and 5-HT2A receptor in myopia induced by flickering light in guinea pigs.

**Methods:**

Forty-five guinea pigs were randomly divided into three groups: control, form deprivation myopia (FDM) and flickering light induced myopia (FLM) groups(n = 15 for each group). The right eyes of the FDM group were covered with semitransparent hemispherical plastic shells serving as eye diffusers. Guinea pigs in FLM group were raised with illumination of a duty cycle of 50% at a flash frequency of 0.5Hz. The refractive status, axial length (AL), corneal radius of curvature(CRC) were measured by streak retinoscope, A-scan ultrasonography and keratometer, respectively. Ultramicroscopy images were taken by electron microscopy. The concentrations of 5-HTin the retina, vitreous body and retinal pigment epithelium (RPE) were assessed by high performance liquid chromatography, the retinal 5-HT2A receptor expression was evaluated by immunohistofluorescence and western blot.

**Results:**

The refraction of FDM and FLM eyes became myopic from some time point (the 4th week and the 6th week, respectively) in the course of the experiment, which was indicated by significantly decreased refraction and longer AL when compared with the controls (*p*<0.05). The concentrations of 5-HT in the retina, vitreous body and RPE of FDM and FLM eyes were significantly increased in comparison with those of control eyes (both *p*<0.05). Similar to FDM eyes, the expression of retinal 5-HT2A receptor in FLM eyes was significantly up-regulated compared to that of control eyes (both *p*<0.05). Western blot analysis showed that retinal 5-HT2A receptor level elevated less in the FLM eyes than that in the FDM eyes. Moreover, the levels of norepinephrine and epinephrine in FDM and FLM groups generally decreased when compared with control groups (all *p*<0.05).

**Conclusions:**

Constant flickering light could cause progressive myopia in guinea pigs. 5-HT and 5-HT2A receptor increased both in form deprivation myopia and flickering light induced myopia, indicating that 5-HT possibly involved in myopic development via binding to5-HT2A receptor.

## Introduction

Myopia is a common refractive error caused by a mismatch between the optical power and the length of the eye with the latter being too long to match the optical power at the given time. The number of myopics keeps growing with increasing pressure from long-period reading, study and usage of computers, especially in Asia and for the present, it has become a social issue drawing enormous attention around the globe. Many experiments have been carried out to explore the underlying pathogenesis of myopia. For this purpose, two classic models have been established in laboratory animals[1] cular form deprivation myopia (FDM), in which an image diffusing goggle or eyelid suture was used to blur retinal image so that ipsilateral eye growth was accelerated and myopia was produced; and 2) lens-induced myopia (LIM), in which spectacle lens was applied in front of retina to shift image plane resulting in compensatory eye growth in an attempt to relocate the retina at the location of the shifted image plane. Although many efforts have been made to treat myopia during the past hundreds of years, however such attempts were only palliative with unclear myopic etiology.

As far as we have known that visual development is not only controlled by genes, but also influenced by the environment. Among diverse environmental factors around us flickering light (FL) from screens of TV, smart phones, tablets and even from some toys, which is aimed to catch kids’ attention, is now so common a phenomenon in our daily life that growing interests have been aroused about the effects of flickering light. There is increasing evidence demonstrating that the animals raised in flickering light were significantly more myopic than those in normal light. With the illumination at a flash frequency of 2 Hz, myopia was successfully induced in C57BL/6 mice [2].Later, Di Y et al[3] in a study on guinea pigs in regard to the effects of light of different frequencies (5, 1, 0.5, 0.25 and 0.1Hz)on the refraction and compensation in ocular length, found that flickering light of 0.5Hz had caused a more obvious myopic shift in refraction and more axial elongation than those lights of other frequencies, which was consequently named flickering light induced myopia (FLM). However, mechanisms of myopic shift in response to flickering light have not been fully clarified.

Recently, much new evidence based on laboratory animals and clinical data demonstrated that many retinal neurotransmitters and neuromodulators have been implicated in the development of myopia. Serotonin (i.e.5-hydroxytryptamine, 5-HT) as one of them, is synthesized by discrete types of neurons in central nervous system(CNS). Retina, as an outgrowth of forebrain, therefore is a part of CNS. Convincing evidence has suggested that 5-HT also exists in the mammalian animals’ retina[4,5,6]and has many functions including altering retinal amacrine cell processing, increasing intraocular pressure, constricting ocular blood vessels, and serving as a mitogen[7]. The actions of 5-HT are mediated by different receptors, which are generally categorized into seven families (5-HT1R to 5-HT7R) according to their structural and functional characteristics and can be further divided into at least 14 subtypes. Except for 5-HT3R which is a ligand-gated ion channel[8], all the others are G-protein coupled receptors (GPCRs).

Myopic chickens formed by monocular deprivation with normal light cycles were accompanied with unaltered retinal 5-HT levels[9]. Furthermore, in 1995 Schaeffel F et al[10] found that a single intravitreal injection of reserpine which both depleted 5-HT and dopamine (DA) storage blocked formation of FDM and LIM efficiently. This may imply that 5-HT plays a positive role in the myopia development. The RT-PCR results revealed that 5-HT1A、5-HT2A、5-HT2C、5-HT3 and 5-HT7 receptor subtypes were normally expressed in the retina of rats and the levels of 5-HT7R and 5-HT2ARwerethe highest [11]. The 5-HT2A receptor (5-HT2AR) is one of the subtypes of 5-HT2 receptors (including 5-HT2A, 5-HT2B and 5-HT2C)[12]. Recently, Yang J et al[13]successfully induced LIM in guinea pigs and demonstrated that 5-HT levels in the retina, choroids and sclera in LIM eyes were significantly higher than those in control group and meanwhile 5-HT2AR expression was also significantly up-regulated. These findings strongly suggested that 5-HT was possibly involved in myopic formation by binding with 5-HT2AR. On the other hand, there is general consensus among the researchers that DA modulates development of myopia, which was supported by the findings that the concentration of which decreased in excessively growing eyes[14, 15]. Nevertheless, 5-HT2AR antagonists could reduce increase of DA in the nucleus accumbens resulting from stimulation of the dorsal raphe nucleus. Hence in summary, all these data support the idea that 5-HT may play an important role in the myopic development, which depends on the subtypes of 5-HT receptors involved, especially is related to 5-HT2AR.

Although 5-HT has been the subject for numerous studies, yet until now, little is known about the importance of 5-HT and 5-HT2AR in the development of the new experimental myopia induced by flickering light. In the present study, we assessed 5-HT levels and 5-HT2AR expression in the ocular tissues of guinea pigs with exposure to flickering light and form deprivation. In addition, we also measured concentrations of another two monoamines: norepinephrine (NE) and epinephrine (E), trying to find out the complicated pathogenesis network involved in myopia.

## Materials and Methods

### Animals and Light Conditions

2-week-old male guinea pigs (Cavia porcellus, the English short-hair stock, tricolor strain, n = 45) were obtained from the laboratory animal center at Fudan university in Shanghai, China. These guinea pigs were raised at the temperature of 20–22°C, the relative humidity of 55–65% with free access to sufficient food, water and daily provided fresh vegetables. The treatment and care of the animals were consistent with the ARVO Statement for the Use of Animals in Ophthalmic and Vision Research and the animal research in the following was approved by the Local Animal Care and Ethics Committee at Jinshan Hospital of Fudan University, Shanghai, China.

These guinea pigs were raised in specially designed cages (50×40×60 cm with mesh size:1.5×5.0 cm) which were totally covered to ensure complete darkness. Each cage was installed with 4 light-emitting diode tubes (LED, intensity: 600lux, wavelength: 600nm, color temperature: 2700k) at its four upper corners to ensure uniform illumination. The illumination was manipulated via temporal luminance modulation by a function generator (Yinuo Automation Co., LTD, Changsha, China; linear output, analogue signal, alternating-current pulse 220 V) linked to those LEDs. All the equipments were under the control of a timer. The LEDs were turned on at 6:00 AM and switched off at 6:00 PM.

The guinea pigs were randomly assigned to three groups: control group(n = 15), form deprivation myopia group (FDM, n = 15) and flickering light-induced myopia group(FLM, n = 15). Animals of the FLM group received the illumination by the square-wave flickering light with a duty cycle of 50% at a flash frequency of 0.5 Hz (i.e. 1 second of dark phase / two seconds) for 8 weeks. During each flicker cycle, luminance varied between 600 lux and 0 lux. In FDM group, monocular deprivation was established by using translucent plastic diffusers made from white moldable plastic glued to guinea pigs’ right eyes. These diffusers were attached far away enough from the eyelid to avoid interference with the normal functions of eyelids. The transmission rate of eye diffusers for light was 60%. The plastic shells were checked at least twice daily to ensure proper attachment to the right eyes and if any of these diffusers became loosened re-attachment was applied. Different from the illumination arrangements for FLM group, the animals of control group and FDM group were maintained with regular illumination of 300 lux with a cycle of alternate12-h illumination (6:00 AM~6:00 PM) and 12-h darkness to match FLM group in total luminance.

### Optical and Biometric Measurements

All measurements were performed at 2-week intervals, i.e. before the treatment started (marked as “0 week”) and after 2, 4, 6 and 8 weeks of treatment, respectively, by a research optometrist helped by an assistant on the condition that the identities of different groups were anonymized to them.

For the measurement of refractive errors, the animals were only controlled by grabbing their bodies without anesthesia. One drop of tropicamide ophthalmic solution (1 mg/ml) was administered every 5 minutes up to 4 times to achieve an optimal cycloplegia and a completely dilated pupil before all the optical measurements. The refractive errors were examined by streak retinoscope at a working distance of 67cm in a dark environment with usage of lens bars to neutralize the two principal meridians. Each of the measurements was repeated several times(more than 3 times). The ultimate refraction of each eye was obtained by averaging the three sets of measurements and expressed as spherical equivalents. Before the measurement of AL, guinea pigs were locally anesthetized by 0.4% oxybuprocaine hydrochloride eye drops (Santen Pharmaceutical Co., Ltd.). The axial length (AL) of each right eye was measured by a scan ultrasonography (11 MHz; SW1000, Suowei Co., LTD, China) and the ultimate AL was the average of five independent measurements. The corneal radius of curvature (CRC) was measured in alert guinea pigs with a keratometer (OM-4; Topcon, Tokyo, Japan)combining with a +8.0 D aspherical lens on the anterior surface of keratometer and the definitive CRC was the mean of the horizontal and vertical measurements, which were measured together for three times by keratometer. A set of stainless steel ball-bearings was used for calibration and the corneal radius of curvature was determined by the mean of three readings on the balls[16].

### Electron Microscopy

Electron microscopy: Sections for transmission electron microscopy were prepared the way previously described by other researchers[17]. Guinea pigs were deeply anesthetized by pentobarbital and the anterior segments of eyes (including cornea, iris and crystalline lens) were removed. Then the remnants of eyeballs were fixed in 2.5% glutaraldehyde and 1% osmium tetroxide solution for at least 48 hours. A 1.5mm diameter trephine set at 0.5mm away from the temporal margin of the optic disc was used to punch out a tissue sample (containing retina, choroid and sclera). Outer retinal morphology was examined and photographed with transmission electron microscope (CM-120, Philips, Netherlands), which was evaluated by a pathologist who did not know the group identity.

### Highperformance Liquid Chromatography (HPLC)

Animals were killed with CO_2_ at the end of the eighth week. Their vitreous, retina, and RPE were dissected from the eyecups carefully under a dissecting microscope and quick frozen in liquid nitrogen. These samples were later homogenized with 0.1 N HClO4 containing 0.1% sodium metabisulfite to prevent the oxidation of catecholamine and the levels of 5-HT, NE and E were measured by HPLC as follows: The samples’ supernatants were injected into an Acclaim C18 column (2.2 μm, 2.1 × 100 mm; Thermo Fisher Scientific) at 38°C. Separations were performed at a flow rate of 0.2 ml/min by using a mobile phase of phosphate buffer containing 0.05 EDTA, 1.7 orthosilicic acid (OSA), 90.0 Na2HPO4, 50.0 citric acid(all concentrations were expressed in mM). The voltage of detection cell side was set at +700 mV with the voltage of guard cell side set at +750 mV. The data collected were analyzed by ChemStation (Agilent Technologies). Peaks and relative concentrations were identified in comparison with the known external standards.

### Immunohistofluorescence

#### 1. Preparation of frozen slides

Guinea pigs were deeply anesthetized with pentobarbital, perfused transcardially with physiological saline and followed by 150–200 ml 4% paraformaldehyde in a 0.1 M phosphate buffer (PB, pH 7.4). Then the eyes were enucleated quickly and the anterior segments of the eyes as mentioned above were removed. The remnants of the eyecups were immediately fixed in fresh 4% paraformaldehyde with 0.1 M phosphate buffer (PB, pH 7.4) for 20 minutes, sequentially cooled in 10%, 20% and 30% sucrose (wt/vol) with 0.1 M PB at 4°C, embedded in optimal cutting temperature compound (OCT; Miles, Elkhart, IN, USA), frozen by liquid nitrogen, then sectioned into a thickness of 15 μm on a cryostat and mounted on gelatin chromium coated slides.

#### 2. Immunohistofluorescence

After blocked in 2% donkey serum with phosphate buffered saline (PBS) and 0.1% Triton (PBST), these slides were first incubated overnight at 4°C in a mixture of rabbit anti-5-HT2A receptors (5-HT2AR) (diluted to 1:200; Abcam; catalogue reference: ab81296), then incubated for another 90 minutes at room temperature with secondary antibody: donkey anti-rabbit Alexa 594 diluted to1:800(Invitrogen, USA; catalogue reference: A21203), rinsed three times in PB, mounted on glass slides and covered with Vectashield (Vector, Burlingame, CA). Images of 5-HT2AR-stained retinal part (one selected region:) were taken by a Leica SP5 confocal laser scanning microscope (Leica, Mannheim, Germany) at the magnification of 63×. And we assessed the positive cells expressing 5-HT2AR in guinea pigs’ retina in one selected field (3000um away from the optic disc) by counting the absolute number of positive cells in this specific field or the optical density which represented the relative number of positive cells. Note five guinea pigs in each group were used for Immunohistofluorescence, 10 sections were harvested from each guinea pigs. The average value of the number of the 5-HT2A receptor positive cells in three randomly selected sections was taken as this guinea pig’s value of 5-HT2A receptor positive cells, then the total average of 5 guinea pigs on the number of positive cells was expressed as means ± SEM and used as the number of 5-HT2A receptor positive cells for each group.

### Western Blot

Guinea pigs were sacrificed and the tissues of retina were quickly collected on ice. Subsequently, retina tissues were homogenized in 2%CHAPS buffer containing10 mM sodiumphosphate, pH 7.2, 1% sodium deoxycholate, 0.15 M sodium chloride and protease inhibitor cocktail and centrifuged at12,000 rpm for 10 min at 4°C. Protein concentration was determined by a MicroBCA protein assay kit(Beyotime Biotechnology, China).Tissue homogenates (50μg protein equivalent each) from the entire retina of each guinea pig was boiled at 100°C in sodium dodecyl sulfate (SDS) sample buffer for 5 mins, electrophoresed on 10% SDS-polyacrylamidegel, and transferred to the polyvinyldifluoridine membrane (Bio-Rad).Then these membranes were blocked with 5% nonfat dry milk in0.1% Tween 20 (TBS-T; 2 mmol/L Tris-HCl, 50 mmol/L NaCl,pH 7.5) for 2 hours at room temperature. Next, it followed by overnight incubation of these membranes with the monoclonal rabbit anti-5-HT2AR antibody (Ab85496) at a 1:1000 dilution at 4°C in the blocked buffer. After that, membranes were washed with 0.1% Tween20, and then treated with goat anti-rabbit IgG conjugated to alkaline phosphatase(1:5,000) for 1 hour at 37°C. Stripping filters and reprobing for GAPDH were carried out fornormalization. Controls for nonspecific binding were determined by omission of the primary antibody. Films were scanned with a film scanner (Image Master VDS; Amersham Biosciences Inc., Piscataway,NJ) and subsequently analyzed to measure optical densities of immunostained bands on the film using an image-processing and analysis system (Q570IW; Leica). The faintest band on each image was assigned a value of 1 and all the other bands were automatically endowed with the relative values compared to the faintest band.

## Data Analysis

Except for those regarding refraction measurement, only the data concerning the right eyes were in consideration and used for further analysis in this study. The alterations of all parameters were obtained by the difference between the data measured on the ‘0’ week and those on the 8th week. Parametric statistical analyses were performed on SPSS Statistics 20 (SPSS, Inc., An IBM Company, based in Chicago IL) and GraphPad Prism 5 (GRAPHPAD Software, Inc., San Diego, CA). Multivariate repeated measurement analysis of variance was applied to compare the refraction, AL and CRC among different groups, Bonferrioni was used to determine the significance of differences between means. A one-way analysis of variance was used to compare the neurotransmitters and 5-HT2AR expression among different groups. The results were expressed as means ± SEM and *p*value<0.05 was regarded as the standard for statistical significance.

## Results

### Optical and Biometric Measurements

It was proved that before establishment of myopia(i.e. on the ‘0’ week), the refraction, AL and CRC of all guinea pigs in three groups showed no differences and so did those data between both eyes of the guinea pigs.

#### 1. Refraction (Fig 1)

**Fig 1:**
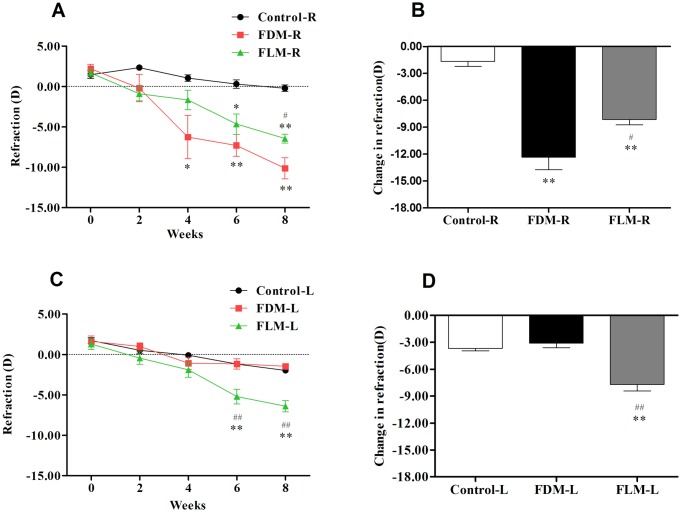
The dynamic status and changes of refraction of guinea pigs in control, FDM and FLM groups. (A)In comparison with control group, significant myopic shift in the right eyes in FDM and FLM group appeared from the 4th week and the 6th week, respectively. (B) In the right eyes when compared with control group, significant changes in the refraction were proved in both FDM and FLM groups. (C)In comparison with control group, there was no significant myopic shift in the left eyes of FDM group whereas significant myopic shift in the left eyes of FLM group was established starting from the 6th week. (D) In the left eyes significant changes in refraction were found in FLM group when compared with either control group or FDM group. (FDM: Form-deprived myopia group; FLM: Flickering light induced myopia group; R: right eyes; L: left eyes. Error bars denote standard errors. (*p<0.05)compared with control group; **p<0.001 vs control group; ^#^*p*<0.05 compared vs FDM group; ^##^*p*<0.001 vs FDM group).

Fig 1 showed the mean and alterations of spherical equivalent refraction for both eyes of the three groups over 8 weeks. From Fig 1A, the right eyes of FDM group and FLM group started to show significant myopic shift from the 4th week and the 6^th^week, respectively (FDM vs control: *p* = 0.008, <0.001, <0.001 for the 4th, 6th, 8th week, respectively; FLM vs control: *p* = 0.004, <0.001 for the 6th, 8th week, respectively), and the refraction of FDM right eyes was significantly reduced than that of FLM right eyes from the 8th week (*p* = 0.025). It was statistically analyzed that the refractive changes of the right eyes in control, FDM and FLM groups were -1.67±0.54D, -12.34±1.42D and -8.14±0.60D, respectively, over 8 weeks’ experiments(Fig 1B). The refractive changes of any two of these three groups all showed significant difference (Fig 1B, FDM vs FLM: *p* = 0.003; FDM vs control:*p* = 0.000; FLM vs control:*p* = 0.000). On the other hand, when considering the left eyes, FLM group became more myopic than the other two groups from the 6th week (Fig 1C, p<0.001 for all). However, no significant differences were demonstrated between the FDM contralateral eyes (i.e. the left eyes) and control left eyes at all-time points (Fig 1C, *p*>0.05). The refractive variation of the left eyes of FLM group(-7.70±0.74D) was significantly higher than that of FDM contralateral eyes (-3.09±0.51D) and the control left eyes (-3.68±0.27D) (Fig 1D, both*p* = 0.000).

#### 2. Axial length (Fig 2)

**Fig 2:**
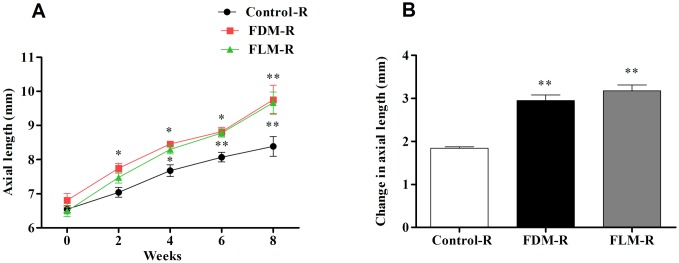
The dynamic status of axial length of guinea pigs in control, FDM and FLM groups. (A) The axial lengths kept elongating in three groups with passage of time during the experimental period. The axial lengths of FDM and FLM eye were significantly longer than that of control eyes from the 2nd week(both *p*<0.05). (B) Over the 8-week experiment, changes in axial lengths were greater in FDM and FLM eyes than those in control eyes (both *p*<0.001) while significant difference was not found between FDM and FLM group. (*p<0.05 vs control group; **p<0.001 vs control group; data were expressed as mean ± SEM).

As for the right eyes, when compared with control group, significant differences in mean axial length in FDM and FLM group started from the 2th week and the 4th week, respectively (Fig 2A, FDM vs control: *p* = 0.005, 0.001, 0.001, 0.000 for the 2th, 4th, 6th, 8th week, respectively; FLM vs control: *p* = 0.048, 0.003, 0.000, 0.000 for the 2th, 4th, 6th, 8th week, respectively)but there was no significant difference between FDM and FLM group (*p*>0.05). Further, the variations of axial length over 8 weeks’ experiment, both FDM and FLM group displayed significant differences in comparison with control group (Fig 2B, both *p* = 0.000)whereas, there was no significant difference between FDM and FLM group (Fig 2B, *p* = 0.191).

#### 3. Corneal radius of curvature (CRC) (Fig 3)

**Fig 3:**
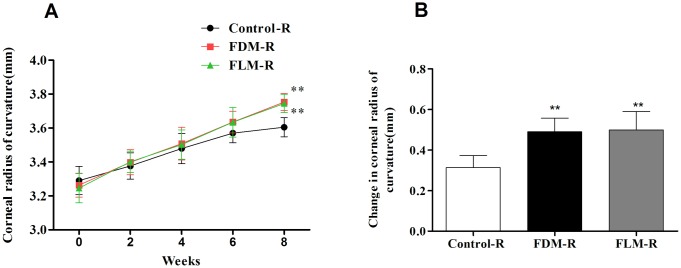
Corneal radius of curvature (CRC) in control, FLM and FDM groups over the 8-week experiment. (A) CRC of the right eyes in three groups throughout the examined period; Compared with control eyes, CRC of the right eyes in FDM and FLM group became greater at the 8th week (both*p<0*.*001*). (B) The CRC alterations in the right eyes of three groups by the end of the experiment; No significant difference was found between FDM and FLM groups (*p>0*.*05*), but both groups have significant difference when compared to the control group (both*p<0*.*001*). (**p<0.001 vs control group; data were expressed as mean±SEM).

With regard to the right eyes, no intergroup differences in CRC were found until the end of the experiment, i.e. the 8th week (Fig 3A).The CRC of eyes in FLM group and FDM group were significantly larger than that of control group (both *p* = 0.000).Furtherly, variations of CRC were significantly obvious in FDM and FLM groups compared to that of control group (Fig 3B, both *p* = 0.000).

### Ultramicroscopy Images of the retina (Fig 4)

**Fig 4:**
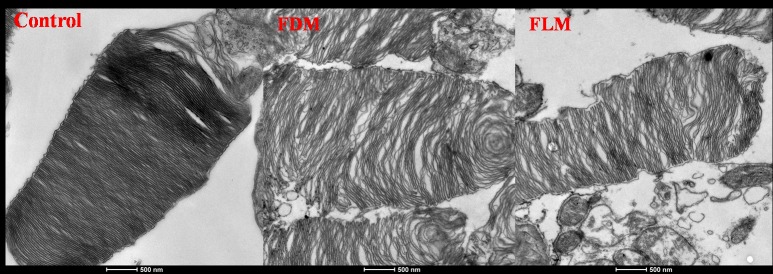
The transmission electron microscope photographs of the disc membranes in three groups. Morphologically, in comparison with control group, both FDM and FLM groups displayed several abnormalities: membrane discs of outer segment of photoreceptors were apparently disordered and the gap between membrane disc became widen, when compared with the control group (scale bar: 500nm).

Under transmission electronic microscope, rough comparison with the image of control group, general abnormalities in the photoreceptors of the retina were found in both FDM and FLM groups and there were no significant differences between them. On closer examination, it was obvious that in both FDM and FLM groups the membrane discs of the outer segment of rod cells were remarkably disrupted, the gap between adjacent membrane discs became widened, but no other striking morphological changes were demonstrated in other fundal layers among these three groups.

### The HPLC measurement of Neurotransmitters Concentrations

At the end of the experiment (on the 8th week), the concentrations of 5-HT, NE and E in fundal layers (retina, vitreous body and RPE) were measured and compared among all these three groups.

#### 1. 5-HT (Fig 5)

**Fig 5:**
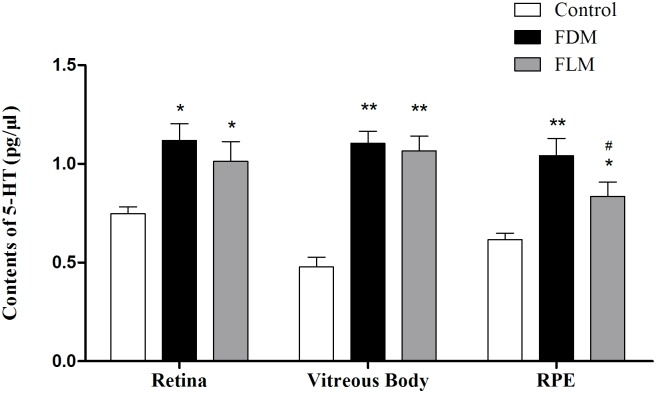
The concentrations of 5-HT contained in the retina, vitreous body and RPE of guinea pigs in different groups after 8 weeks’ experiment. Treatments in FDM and FLM groups resulted in a significant increase in 5-HT concentrations all three parts in comparison with corresponding part in control group (all *p*<0.05). (*p<0.05 vs control group; **p<0.001 vs control group;^#^*p*<0.05 vs FDM group; data were expressed as mean ± SEM).

From Fig 5, significantly higher concentrations of 5-HT in both FLM and FDM group were detected in all the tissues (retina, vitreous body and RPE) at the end of the experiment when compared with control group (retina, vitreous body and RPE of control group vs those tissues of FLM: *p* = 0.015, 0.000, 0.024, respectively; retina, vitreous body and RPE of control group vs those of FDM: *p* = 0.005, 0.000, 0.000, respectively). More intriguingly, 5-HT concentration of FLM group was significantly lower in the layer of RPE than that of the same layer of FDM group (*p* = 0.027) whereas no significant difference of 5-HT was demonstrated in the tissues of vitreous body(*p* = 0.561) and the retina (*p* = 0.299) between FLM and FDM group.

#### 2. NE (Fig 6A)

**Fig 6:**
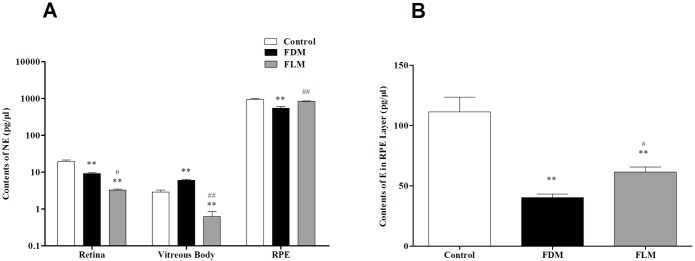
The concentrations of NE and E in fundal layers of guinea pigs in different groups. (A) NE level was significant lower in FLM eyes than that in control and FDM eyes in the part of retina and vitreous body (all *p*<0.05). (B) The level of E in the part of RPE in FLM group was lower than that in control groups, but higher than that in FDM groups (both *p*<0.05). (*p<0.05 vs control group; **p<0.001 vs control group;^#^*p*<0.05 vs FDM group; ^##^*p*<0.001 vs FDM group; data were expressed as mean ± SEM).

Generally, NE concentrations of the retina and vitreous body were much lower than that of the RPE in all eyes. In the retina, in comparison with control group, significant decrease in NE concentration was proved in both FDM and FLM groups(both *p*<0.001). And in the vitreous body, when compared with control group there was significant reduction in NE level in FLM group while significant increase in FDM group (both *p*<0.001).Therefore, significant differences in NE in retina and vitreous body were observed among all the three groups (*p*<0.05). Furthermore, in RPE layer, the concentration of NE was significantly lower in FDM group than both control and FLM groups and there was no significant difference between control and FLM groups(FDM vs FLM: *p*<0.001; FDM vs control: *p*<0.001; FLM vs control: *p* = 0.088).

#### 3. E (Fig 6B)

With the amount of E in the retina and vitreous body being too low to be detectable, so only the concentration of E in RPE layer was analyzed. The average levels of N in control, FDM and FLM groups were 111.29±12.19 pg/μl, 40.31±2.99 pg/μl and 61.48±4.11 pg/μl, respectively. Remarkable differences were found between any two groups (all *p*<0.05).

### 5-HT2A Receptors Expression (Figs 7and 8)

**Fig 7:**
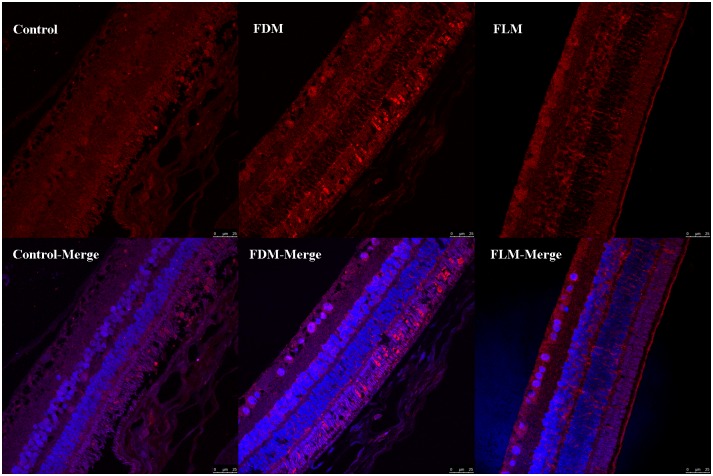
Immunofluorescene photographs of retina obtained by staining 5-HT2A receptors (5-HT2AR) and nuclei. Red fluorescence represented 5-HT2AR, and blue represented cell nucleus (scale bar: 25μm). Up-regulation in the expression of 5-HT2ARweremainly found in the layer of retinal ganglion cells(RGCs), inner nuclear layers (INL) and photoreceptors after in both FDM and FLM group.

**Fig 8:**
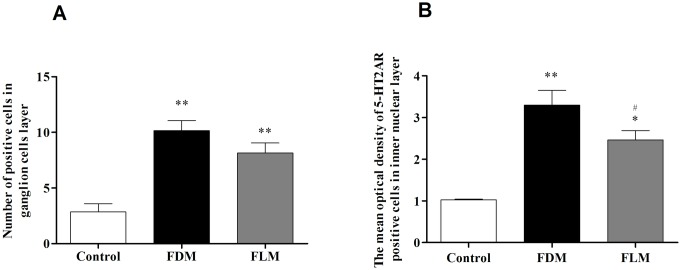
The retinal levels of 5-HT2AR in three groups. (A) The number of positive cells in retinal ganglion cells(RGCs) of both FDM and FLM eyes increased significantly compared with the control group (*p*<0.001). (B) The average optical density of 5-HT2AR positive cells in inner nuclear layers (INL) was calculated for the three groups, respectively. In the layer of INL, the number of 5-HT2AR increased both in FDM and FLM groups, significant differences were detected among these three groups (*p*<0.05) (*p<0.05 vs control group; **p<0.001 vs control group;^#^*p*<0.05 vs FDM group; all data were showed as mean±SEM).

Fig 7 demonstrated the images of all layers of retina obtained by immunofluorescence technique, from which it was obvious that the 5-HT2AR-expressing cells were mainly distributed in the layers of RGCs, INLs and photoreceptors/RPE. Moreover, the expression of 5-HT2AR in the layer of INL of control, FDM and FLM eyes was compared by both absolute count of the positive cells (Fig 8A) and the relative amount of the positive cells expressed as the mean optical density (Fig 8B).As indicated by Fig 8A, in comparison with control group, both FDM and FLM group showed significantly higher count of the positive cells (both *p*<0.001) whereas no significant difference was found between FDM and FLM group(both *p* = 0.116). In the aspect of the mean optical density of the positive cells, the expression of 5-HT2AR was significantly higher in FDM and FLM group when compared with control group(FDM: *P*<0.001 vs the control group; FLM: *P* = 0.001 vs the control group). Meanwhile, there was significantly lower expression of 5-HT2AR in FLM group in comparison with FDM group (Fig 8B,*p* = 0.026).

**Fig 9:**
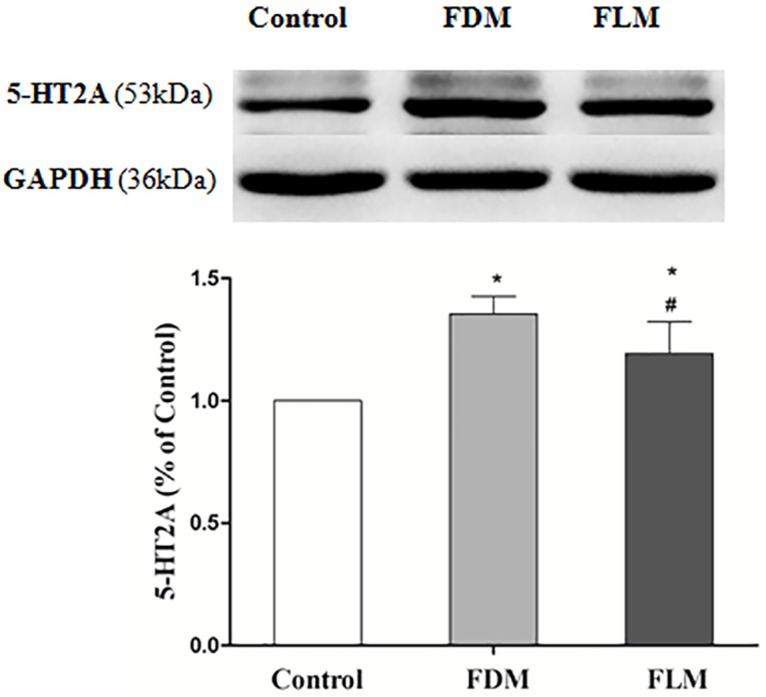
The Western blot results of 5-HT2A receptor expression in three different groups. The 5-HT2A receptor expression significantly increased in different degrees in FDM and FLM groups when compared with control group, in which the FDM group increased the most. (*p<0.05 vs control group; ^#^*p*<0.05 vs FDM group; all data were showed as mean±SEM).

Subsequently, the expression of 5-HT2ARwere investigated by using Western blot. As shown in Fig 9, western blotting analysis showed that 5-HT2AR level increased in FDM and FLM groups (both *P* = 0.001 vs the control group). Note that 5-HT2AR expression increased significantly less in FLM group than that in FDM group (*P* = 0.005).

## Discussion

From the results explained above, it was revealed that the refractive error and axial length of the eyes in FLM group changed quickly towards to a myopic shift and so did FDM group. Morphologically, the abnormalities in the membrane discs of the outer segment of rod photoreceptors in the myopic eyes were shared by both FDM and FLM groups. All these findings supported that not only form deprivation but also flickering light of 0.5Hz could induce myopia in guinea pigs.

There is growing evidence to show that light parameters, such as intensity, wavelength and frequency were important for the visual development. It has been proved that the intensity of light can affect refractive development in chicks with low intensities inducing myopia and high intensities inducing hyperopia by Cohen Y[17]et al in their study. In another study on biometric measurements by Wang [18] et al, guinea pigs raised in 530nm green light had a myopic refractive status. Besides, Schwahn HN[19]revealed that flickering light could suppress lens-induced as well as deprivation induced refractive errors in chickens, which was mainly attributed to corneal flattening. In contrast, Crewther SGet al[20] showed that chicks wearing positive, plano or negative lens raised under the condition of flickering light of low frequencies (1, 2 and 4 Hz) ended up with a general myopic shift while little refractive change in the ones without lens, which was explained by the hypothesis that flash of low frequency could lead to fluid increase in the anterior chamber. Based on all these studies, work of Di Y et al concluded that chronic exposure to flickering light of 0.5and 5Hz altered emmetropization in guinea pigs’ eyes and flickering light of 0.5Hz was more effective than 5Hz as to myopic induction, which was similar to an earlier finding that myopia could be induced in B6 mice by flickering-light of 2Hz. Therefore, our research is in line with these previous studies and yet again proves that myopic shifts can be induced by flickering light of low frequency like 0.5Hz.

5-HT (serotonin) is an important neurotransmitter involved in many physiological functions, especially those occurring in the brain. Embryologically retina is an outgrowth of the central nervous system. So no wonder, many previous studies have presented evidence that 5-HT was also a neurotransmitter in the retina[21], which was further proved by that5-HT receptors were present in rats’ retinal pigment epithelium. Diverse functions of 5-HT are mediated by a variety of specific receptors. Until now 5-HT1 to 5-HT7 have been recognized, but researchers have different opinions on the role of 5-HT in myopic development. Schaeffel F et al reported that 5-HT may be involved in the negative lens-induced myopia since inhibition of negative lens-induced myopic shift in axial length was accompanied by a drop in 5-HT levels and even the disappearance of serotonergic cells in the retina. Whereas, the data of George A[7]demonstrated that the number of 5-HT-containing amacrine cells have increased in LIM eyes suggesting that serotonin had a stimulatory role in LIM, although high doses of serotonin were inhibitory. In our study on guinea pigs, differed from the result that 5-HT levels were unaltered in FDM chickens, it showed that 5-HT concentration increased in both FDM and FLM group, which was similar to the research of Yang J et al, in which it was demonstrated that 5-HT levels in the retina, choroid and sclera of LIM eyes were significantly higher than those of control group. Considerable species differences or different experimental methods may contribute to these inconsistencies. A striking observation in our study was that dramatic differences in 5-HT concentration among FDM, FLM and control group were found in RPE layer. Since RPE lies next to the layer of photoreceptors of neural retina and serves as a transport passage between choriocapillaries and the photoreceptors, it was hypothesized that different mechanisms underlying form deprivation and flickering light stimulation caused myopia.

Furthermore, we have measured the expression of 5-HT2AR in the retina on the basis of previous researches which had demonstrated the presence of 5-HT2AR in the retina[22–24]. In our study, up-regulated 5-HT2AR expression was observed in guinea pigs’ eyes after form deprivation or exposure to flickering light. Grewal JS et al[25] indicated that 5-HT2AR appears to mediate the mitogenic effect in renal mesangial cells. In most cases, the structural cause of myopia is an excessive axial length of the eye, which was influenced by scleral remodeling. Hence, 5-HT may be involved in myopic process by binding to 5-HT2AR to strengthen scleral remodeling. Meanwhile, there was some evidence suggesting that 5-HT system could interact with DA system and several different 5-HT receptor types have been implicated in this process[26, 27]. There was strong evidence that DA played a critical role in the development of myopia, which decreased during the process of myopic formation in animal models[28, 29] and there was also the evidence of serotonergic inhibition of DA in the brain[30,31]. Hence, associating these findings together, it’s possible that increased 5-HT levels may cause a down-regulation of DA in the retina, which could be proved by that in the nucleus accumbens, 5-HT2AR antagonists could reduce increase in DA[32]and the previous study of interaction between 5-HT and DA by Zifa E[33]and Kato S[34]. All these research findings will favor the expectation that actions of 5-HT in myopic regulation are mediated by 5-HT2AR with interaction with DA, directly or indirectly. In addition, other possibilities were also raised by some researchers that 5-HT’s actions on the refractive status and ocular elongation involved increased intraocular pressure (IOP), vascular modifications in the retina and choroid and mitogenic effects. Hence further studies are needed to focus on identifying the relationship between 5-HT and DA in signal pathways and the exact mechanism underpinning the role of 5-HT in myopic formation.

Apart from 5-HT, there are also some reports suggesting that both NE and E are the neurotransmitters in the retina[35–37] and previous evidence proved that NE and E represented approximately 5% of the DA concentration[38].But few researches have been performed to investigate the alterations in the levels of NE and E in myopia development. So in our experiment, HPLC was used to measure the concentrations of these two neurotransmitters. As for the concentration of NE, much lower levels in the retina and vitreous body were observed and the retinal NE levels of FLM eyes showed significant decrease in comparison with both FDM and control eyes. However, different trends in the alteration of NE were found in vitreous body and RPE between FDM and FLM groups. These data revealed different mechanisms related to NE may exist between FDM and FLM. On the other hand, the presence of E was only detected in RPE layer in guinea pigs. E is derived from NE by catalysis of phenylethanolamine N-methyltransferase (PNMT), which has been detected in the retinas of various vertebrate species[39]. Compared with control eyes, we found that E concentration in the RPE layers of FDM and FLM eyes both showed a dramatic decrease after 8 weeks’ experiment. Generally, the results in the present study more or less showed a decrease in both NE and E contents with experimental myopia. Based on the alterations in the concentrations of NE and E, we speculated that NE and E may also played important parts in myopia development in guinea pigs’ myopia induced by form deprivation or flickering light stimulation and the effects of 5-HT and monoamines (NE and E) may exert through different mechanisms.

## Conclusions

The Flickering light is effective and useful in the information of experimental myopia. Similar to FDM, FLM displayed significant increase in the concentrations of 5-HT in the retina, vitreous body and RPE, up-regulation of 5-HT2AR in the retina and general decrease in the concentrations of NE and E, which further suggests the involvement of 5-HT, 5-HT2AR, NE and E in the myopia development. Hence, a better and in-depth understanding of the roles of 5-HT, 5-HT2AR, NE and E in flickering light induced myopia is required.

## Supporting Information

S1 Appendix More relevant data underlying the findings described in manuscript. (ZIP)
