# Supplementary figures and images for: Effects of Constant Flickering Light on Refractive Status, 5-HT and 5-HT2A Receptor in Guinea Pigs
Source: PLoS One. 2016 Dec 13;11(12):e0167902. doi: 10.1371/journal.pone.0167902 (PMC5154534; doi:10.1371/journal.pone.0167902)

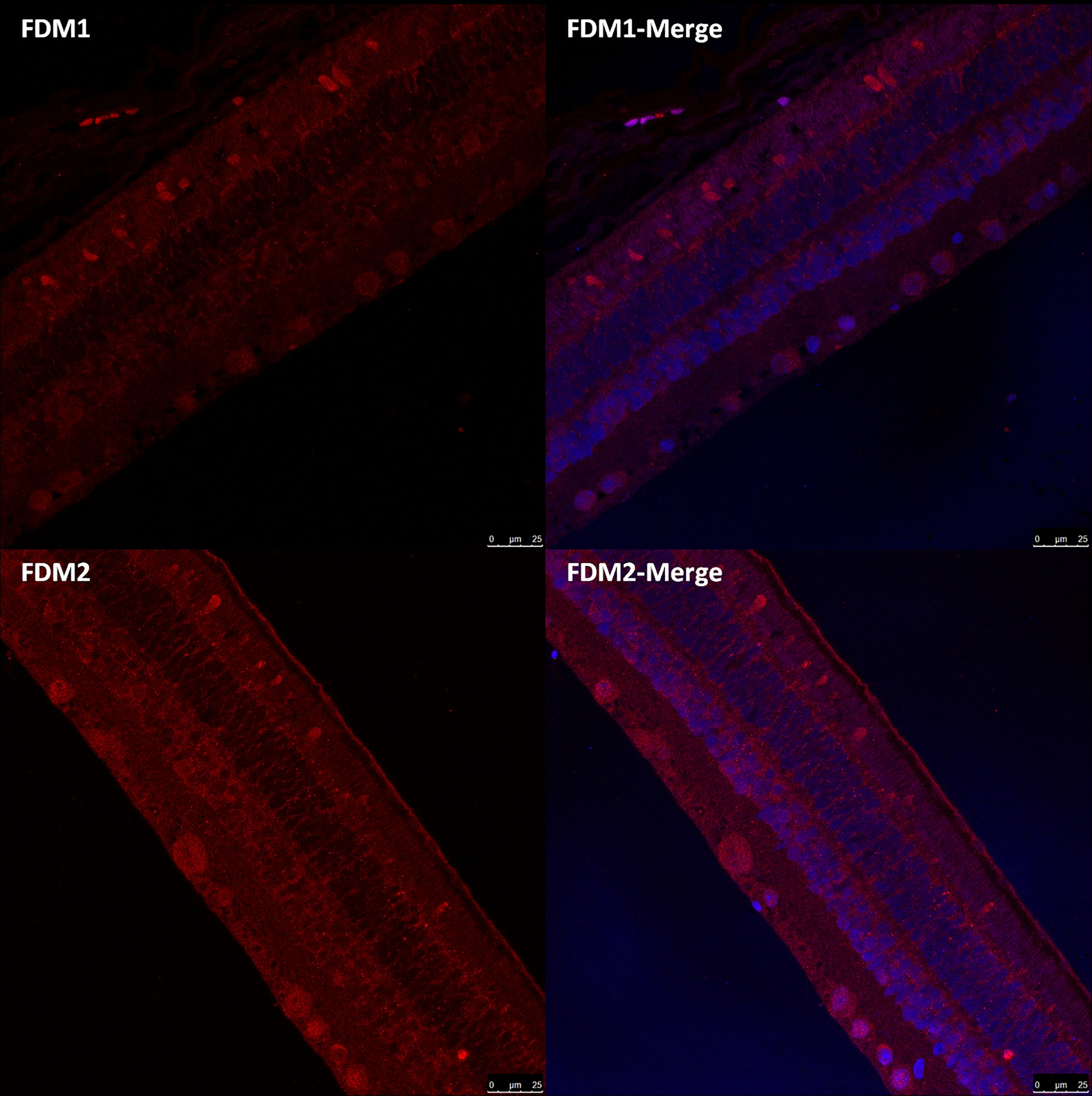

Supplement: S1 Appendix — (ZIP) [file pone.0167902.s001.zip › More Immunofluorescence Results (FDM).tif]

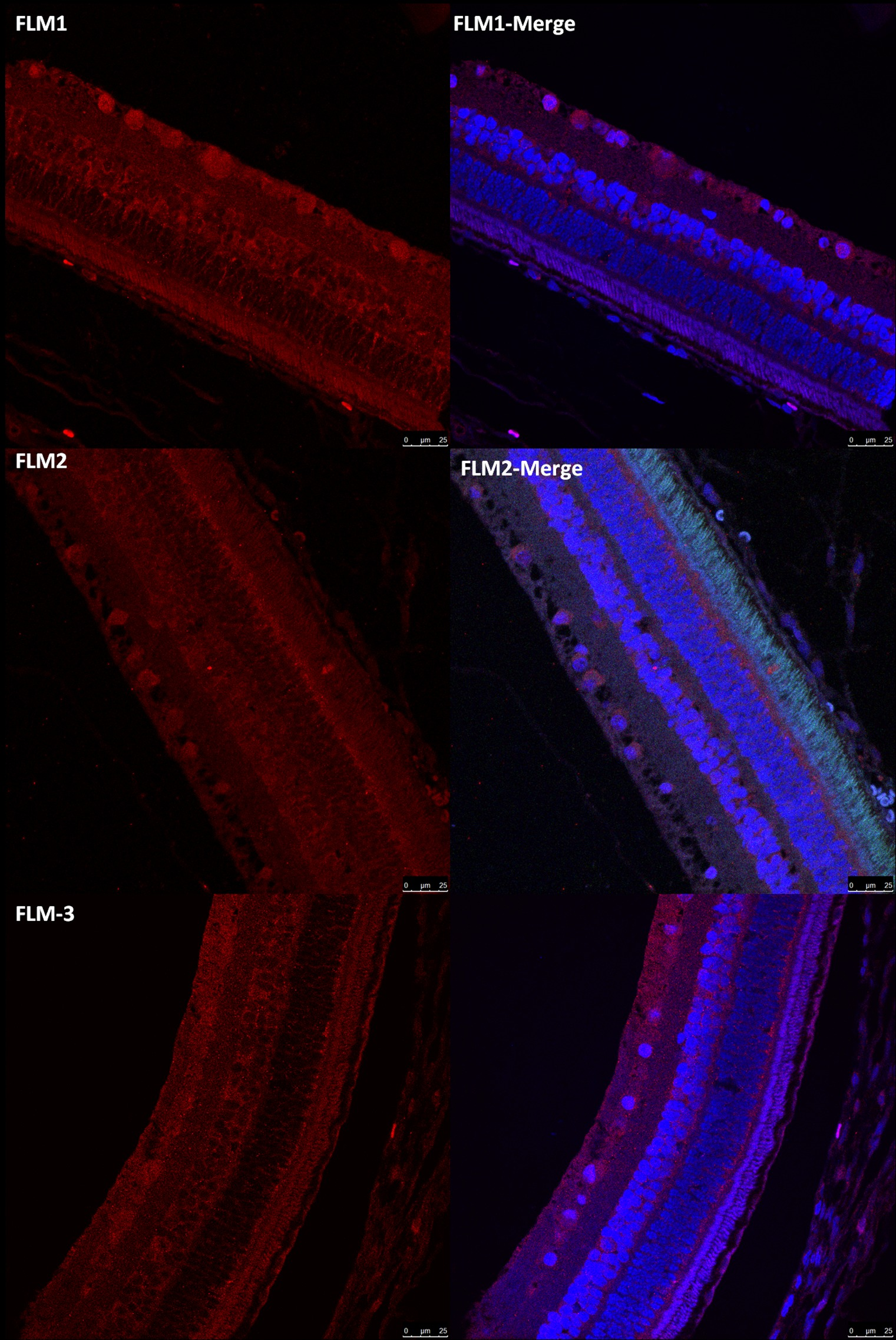

Supplement: S1 Appendix — (ZIP) [file pone.0167902.s001.zip › More Immunofluorescence Results (FLM).tif]

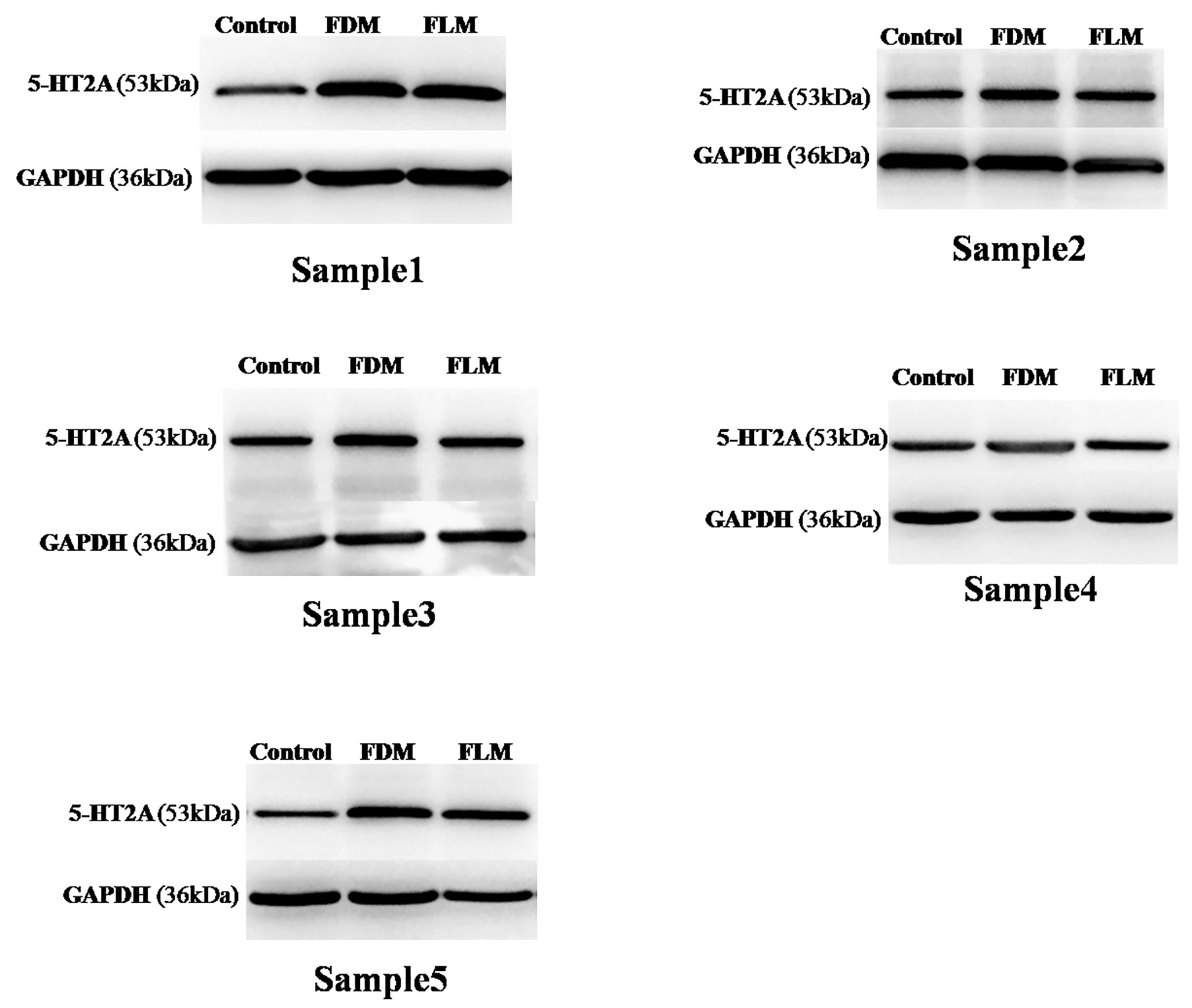

Supplement: S1 Appendix — (ZIP) [file pone.0167902.s001.zip › More Western Blot Results.tif]

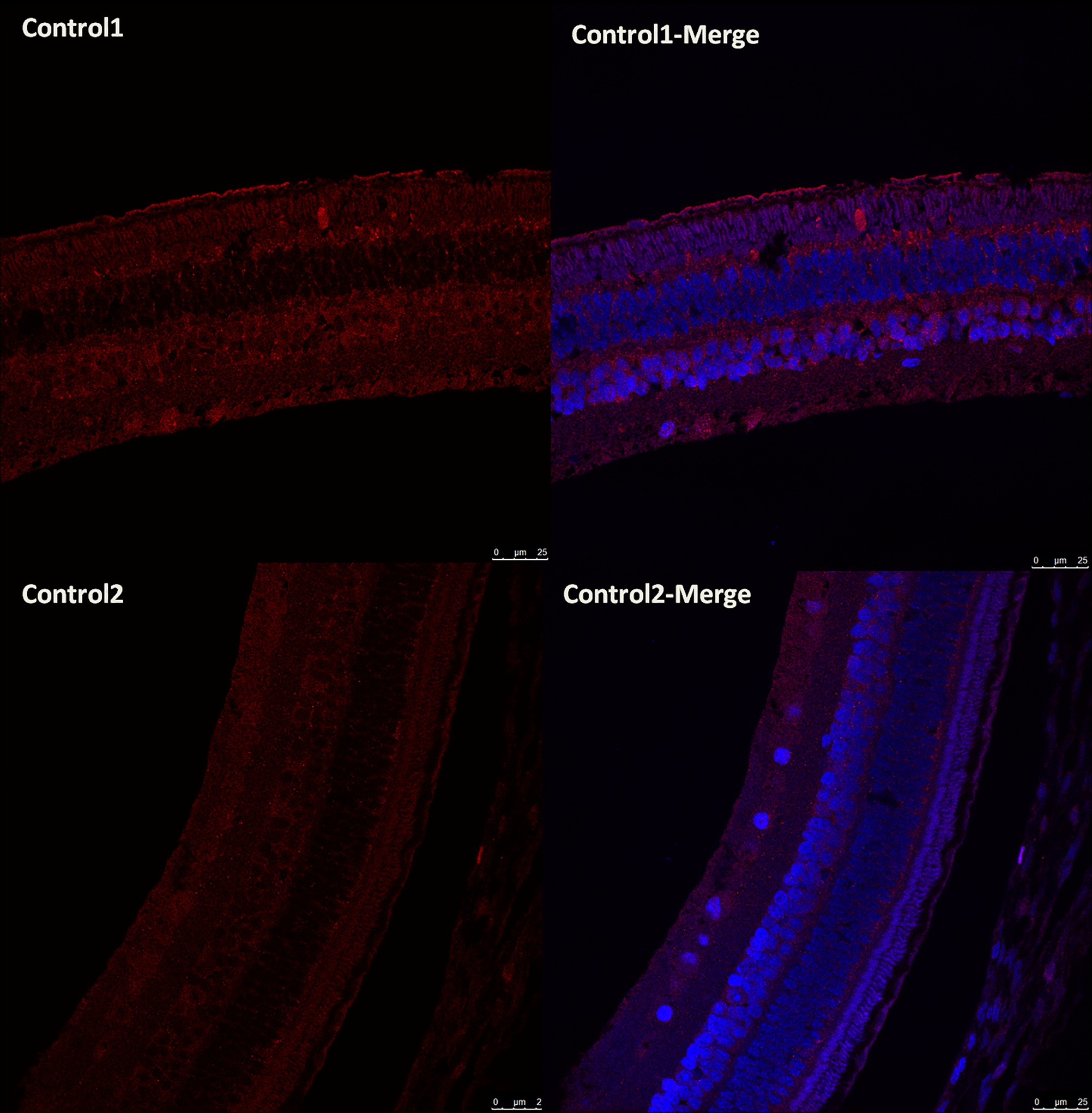

Supplement: S1 Appendix — (ZIP) [file pone.0167902.s001.zip › More Immunofluorescence Results (control).tif]
